# Advances in artificial intelligence to model the impact of El Niño–Southern Oscillation on crop yield variability

**DOI:** 10.1016/j.mex.2025.103650

**Published:** 2025-09-27

**Authors:** Usa Humphries Wannasingha, Muhammad Waqas, Angkool Wangwongchai, Phyo Thandar Hlaing, Porntip Dechpichai, Shakeel Ahmad

**Affiliations:** a Department of Mathematics, Faculty of Science, King Mongkut’s University of Technology Thonburi (KMUTT), Bangkok,10140, Thailand; b The Joint Graduate School of Energy and Environment (JGSEE), King Mongkut’s University of Technology Thonburi (KMUTT), Bangkok, 10140, Thailand; c Yunnan Provincial Key Laboratory of Soil Carbon Sequestration and Pollution Control, Faculty of Environmental Science and Engineering, Kunming University of Science and Technology, Kunming 650500, China

**Keywords:** El Niño-Southern oscillation, Artificial intelligence, Machine learning, Deep learning, Cereal crops, Crop yield

## Abstract

El Niño-Southern Oscillation (ENSO) has a significant impact on global agricultural systems in tropical regions, where rainfed rice production is highly vulnerable to climatic extremes, including droughts and floods. This systematic review synthesizes findings from two decades of research to examine the effects of ENSO phases—El Niño and La Niña—on cereal crop yields, with a focus on rainfed rice in Thailand. The study also evaluates the role of artificial intelligence (AI) in predicting ENSO-induced impacts on crop productivity.

Findings indicate that El Niño events often reduce rainfall, increasing drought stress, while La Niña leads to excessive precipitation and flooding—both of which adversely affect rice productivity. AI-based studies have shown that models such as Random Forest (RF), Long Short-Term Memory (LSTM), and Convolutional Neural Networks (CNNs) demonstrate strong potential, although limitations remain in terms of scalability and local adaptation.

• Hybrid modeling approaches that integrate physical and statistical methods are essential.

• Future research must enhance data quality and integrate adaptive technologies to support climate-resilient agriculture.

## Graphical abstract

**Figure:**
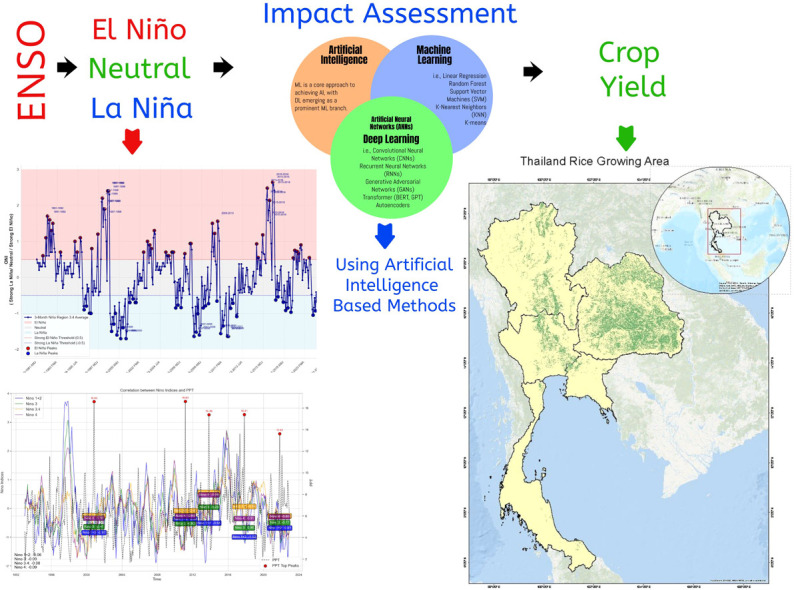

Table 1 Specifications table

**Table:** 

**Subject area**	Engineering
**More specific subject area**	Modeling and Forecasting
**Name of the reviewed methodology**	Artificial Intelligence-Based Techniques
**Keywords**	El Niño-Southern Oscillation; Artificial Intelligence; Machine Learning; Deep Learning; Cereal Crops; Crop Productivity
**Resource availability**	N.A.
**Review questions**	RQ-1: How do different ENSO phases (El Niño, La Niña, and Neutral) influence Thailand’s climate variability and crop productivity? RQ-2: What are the specific effects of ENSO-induced climate variability on rice yields in Thailand? RQ-3: How can artificial intelligence (AI) methods, including machine learning (ML) and deep learning (DL) techniques, be applied to assess the impacts of ENSO on cereal crop production?

## Background

Global warming has become a critical issue in the 21^st^ century [1], driving significant changes in climate variables and initiating long-term shifts known as climate change [2]. This phenomenon exacerbates extreme events, such as prolonged droughts [3], severe floods [4], and landslides [5], with adverse socioeconomic effects [6], as influenced by the El Niño-Southern Oscillation (ENSO) [3]. The ENSO is recognized as a phenomenon characterized by irregularly periodic fluctuations in winds and sea surface temperatures (SSTs) across the tropical eastern Pacific [[3], [4], [5]]. These variations have been observed to impact the climate patterns of an essential part of the tropics and subtropics [6]. The phenomenon characterized by the warming phase of SST is called El Niño, while its counterpart, the cooling phase, is known as La Niña [3]. Both phases can last several months, and typically, they occur at irregular intervals of a few years, with the intensity of each phase varying [4]. It is widely acknowledged that the ENSO plays a significant role in generating inter-annual fluctuations in weather and climate patterns across various regions globally [7]. These large-scale anomalous changes in ENSO-generated climate variability and weather extremes can result in crop failure, food insecurity, famine, and loss of property and life [8].

Monitoring and predicting climate-induced variations in crop yields, production, and export prices in key food-producing regions are critical for ensuring food security in import-dependent countries [9]. The impact of climate events such as El Niño and La Niña on agricultural production, prices, and farmer income is significant [10]. El Niño-induced droughts can result in crop failure due to reduced rainfall, while La Niña’s excessive rainfall can cause flooding and disrupt pest management [11]. ENSO events, with their relatively short duration, substantially impact food crops, which rely heavily on water availability and climatic conditions [12,13]. These disruptions can lead to increased food prices, inflation, and insecurity, particularly for staple crops such as rice and cassava [14]. Research indicates that the effects of ENSO on agricultural productivity vary globally, with El Niño decreasing production in some regions and La Niña benefiting others, influencing economic stability in affected countries [15,16]. Seasonal ENSO forecasts are highly reliable, and linking global yield variations with ENSO phases can significantly enhance food monitoring and famine early warning systems [17]. While the regional impacts of ENSO on temperature, rainfall, and yields are well-documented, there is no comprehensive global map detailing ENSO’s effects on crop yields [18,19].

Existing studies primarily focus on specific regions, such as Australia [20], China [21], the US [22], and other countries, including India [23], Thailand [2], and Africa [24], providing limited insights. This lack of global data complicates the accurate quantification of ENSO’s overall impact on crop yields, where yield variation, influenced by climatic factors, plays a crucial role in production outcomes [[25], [26], [27]]. Conversely, in the US and certain European regions, El Niño is associated with increased economic growth [28].

Agriculture accounts for approximately 25 % of the region’s GDP in Asia and employs about 60 % of the population [29]. Staple crops such as rice, wheat, and maize play a crucial role in human consumption, with Asia producing 90 % of the global rice supply [30]. According to FAO, major producers, including China, India, and Indonesia, achieve high yields due to advancements in mechanization and the adoption of improved seed varieties [31]. Wheat production is similarly substantial, with China and India collectively producing over 300 million tons (Mt) annually [32]. The average yields for rice and wheat in Asia are 4.5 tons per hectare and 3.2 tons per hectare, respectively [33]. These cereals are integral to food security in densely populated nations and contribute to daily caloric intake [34]. Furthermore, agricultural GDP growth is twice as effective in reducing poverty as growth in non-agricultural sectors, underscoring the need to enhance productivity and promote exports [35]. Agriculture remains a dominant sector in Southeast Asian economies, employing over 60 % of the labor force during cropping seasons and contributing approximately 20 % to the GDP, with exports constituting a substantial share [36].

Climate variability due to ENSO in Southeast Asia is critical for agriculture and food security [8,[37], [38], [39], [40], [41], [42]]. Naylor et al. (2007) noted that rice and maize production in Indonesia is highly vulnerable to ENSO-related climate changes [37]. Likewise, Angulo et al. (2009) also found that El Niño has a detrimental impact on rice yield in both irrigated and rainfed ecosystems in the Philippines [39]. Furthermore, Al-Amin and Alam (2021) and Pheakdey et al. (2017) have demonstrated that climate variability associated with El Niño has negative impacts on agriculture in Malaysia, Thailand, and Cambodia [8,40,41].

To understand the regional and local impacts of ENSO, this review uses Thailand as a case study, supporting a detailed analysis of its implications at a localized scale. Climate variability in Thailand is a critical issue [43]. The country’s temperature has risen by 1.04–1.80°C per century, and fluctuations in rainfall have impacted agricultural productivity and income [2]. Since 80 % of Thai farmland depends on rainfall, understanding its spatial and temporal distribution is crucial [44].

Recent studies highlight the complex relationships between ENSO and regional climate variability. Kane (2006) observed a weakened correlation between El Niño and Indian droughts, with only 60 % of events showing effective rainfall links [45]. In Thailand, Bridhikitti (2013) demonstrated that incorporating ENSO/IOD signals into rainfall models significantly improves forecasting compared to aerosol data [46]. Yang and Jiang (2014) found that the NCEP Climate Forecast System (CFSv2) effectively predicts ENSO types and their East Asian climate impacts with higher accuracy for summer and fall [47]. Infanti and Kirtman (2016) showed the NMME system’s ability to forecast North American temperature and rainfall during ENSO events [48]. Abid et al. (2018) reported higher forecast ability for rainfall in the Southwestern Arabian Peninsula during El Niño [49]. Sohn et al. (2019) emphasized residual ENSO variability as a critical factor limiting the predictability of tropical Pacific rainfall [50]. Lastly, Hu et al. (2021) linked ENSO to interannual summer rainfall variability over the Tibetan Plateau [42].

Previous works using mathematical and statistical models [[51], [52], [53]] have provided some understanding of complex relationships between variables; however, these techniques cannot capture non-linear variability [54,55]. The application of artificial intelligence (AI), including machine learning (ML) and deep learning (DL), has improved the evaluation of the ENSO effects on cereal crop production [[56], [57], [58]]. Physical-based dynamical models improve short-range forecasts but fail to capture the stochastic effects of ENSO on cropping systems [59,56]. AI methodologies solve these problems with the help of pattern analysis of extensive data [2]. Support vector machines (SVMs) [60] and decision trees (DTs) [61] are used to predict yield anomalies, but both require significant feature engineering. DL models such as CNNs and RNNs learn high-level features and temporal patterns, enhancing the predictive capability [62]. Probabilistic AI models combined with frameworks such as the Decision Support System for Agrotechnology Transfer (DSSAT) provide early warning systems with longer lead times for ENSO-caused crop losses [63]. However, regional variability and data quality remain key issues that need further development to expand these applications successfully [44,56,62]

Therefore, understanding climate variability by ENSO at a national level and its impact on crop yields is crucial for improving the accuracy of yield forecasting models. This review aims to investigate the predictability of crop yield changes resulting from ENSO at the national level. The objectives of this review are: **(1)** to understand and synthesize the existing literature on the effects of ENSO climate fluctuations on cereal crop yields, particularly rice in Thailand; **(2)** to explore and identify AI methods, including machine learning and deep learning techniques, for assessing the impacts of ENSO on cereal crop production; and **(3)** to identify research gaps and propose recommendations for improving the capacity to address ENSO-related climate risks in agriculture.

## Method details

This systematic review was conducted to evaluate the current state of knowledge regarding the impacts of the ENSO on crop yields, with a particular focus on applying AI techniques. The methodology adhered to established systematic review protocols to ensure transparency, rigor, and replicability.

The literature search was conducted across six major academic databases, including Scopus, IEEE Xplore, ScienceDirect, Web of Science, Google Scholar, and PubMed. The search utilized a combination of keywords and phrases to ensure the inclusion of relevant studies. These terms encompassed ENSO-related expressions such as “El Niño,” “La Niña,” “ENSO,” and “climate variability,” as well as agriculture-related terms such as “cereal crop yields,” “rice production,” “agriculture,” and “Thailand.” Additionally, terms related to artificial intelligence, including “Artificial Intelligence,” “machine learning,” and “deep learning,” were incorporated into the search strategy. Search filters were applied to limit the results to peer-reviewed articles, ensuring the inclusion of high-quality evidence. Conference proceedings, technical reports, and books were also considered to provide a comprehensive perspective on the topic.

Inclusion criteria were established to ensure the relevance and rigor of the selected studies. Eligible publications appeared in peer-reviewed journals, conference proceedings, or technical reports that specifically addressed the effects of ENSO on cereal crops, emphasizing rice production. Studies were required to involve the application of AI techniques, such as machine learning and deep learning, in assessing or mitigating climate-induced yield variability. The review prioritized studies focusing on Thailand or comparable regional contexts. Exclusion criteria were applied to exclude non-peer-reviewed articles, grey literature (excluding technical reports), and studies that did not address the impacts of ENSO on agriculture or lacked an AI methodological focus.

The review was structured around three research questions to guide the synthesis and analysis of findings. The first research question sought to understand how different ENSO phases (El Niño, La Niña, and Neutral) influence Thailand’s climate variability and crop productivity. The second research question examined the specific effects of ENSO-induced climate variability on rice yields, particularly in the context of rice production in Thailand. The third research question investigated the application of AI methods, including ML and DL techniques, in assessing the impacts of ENSO on cereal crop production.

Data was extracted systematically using a structured template to collect information from the selected articles. Extracted data included the objectives of each study, the methodology employed, the geographical focus, the type of crop analyzed, the AI techniques applied, and the key findings reported. The articles were subsequently categorized into thematic areas, including the impacts of ENSO on rice yield in Thailand, the relationship between ENSO phases and crop productivity, the applications of AI and ML in ENSO impact assessments, and the identification of gaps in the existing literature (Table 1). This thematic analysis enabled a comprehensive synthesis of findings under each research question, facilitating a structured comparison of ENSO impacts and AI applications.

**Table 1:** Selected literature based on research questions.

**Research Questions**	**Reviewed Literature**
Understanding of the ENSO phenomenon	[3,4,14,46,56,[64], [65], [66], [67], [68], [69], [70]].
ENSO effects on rice yields in Thailand	[13,21,[71], [72], [73], [74], [75], [76], [77], [78], [79], [80], [81], [82]].
AI methods for assessing ENSO impacts on crop yield	[2,44,56,71,[83], [84], [85], [86], [87], [88], [89], [90], [91], [92], [93], [94], [95], [96], [97], [98], [99], [100], [101], [102], [103], [104], [105], [106], [107], [108]].

## Results

### Understanding of the ENSO phenomenon

The ENSO is a coupled ocean-atmosphere system that is a significant source of climate variability [4]. ENSO oscillates among three phases: the warm phase, known as El Niño; the cold phase, referred to as La Niña; and the neutral state. During El Niño, warm water is displaced eastwards, and equatorial trade winds are weakened, leading to above-average SSTs in the eastern Pacific. On the other hand, La Niña enhances the expected conditions as the trade winds increase their strength and force warm water westward while encouraging cold, nutrient-rich water to well up along the Pacific coast of the Americas [3,4,64]. These oceanic events are in phase with the atmospheric Southern Oscillation, a variation in sea level pressure difference between the western and eastern tropical Pacific Ocean [65,66]. ENSO results from the interactions within the ocean-atmosphere system of feedback. According to Bjerknes, positive feedback starts with SST anomalies that suppress the Walker Circulation and Easterly trade winds. It, in turn, modifies the initial SST anomaly in a way that changes the distribution of heat in the ocean [3]. However, ENSO phases do not last forever because feedback mechanisms, such as wave reflection at the ocean boundaries and changes in ocean heat content, bring back balance and often cause the phase to switch [3,4].

The ENSO cycle is not very regular; events occur every 2-7 years and may last several months to over a year [67]. This variability is due to the interannual interaction of seasonal cycles, stochastic atmospheric fluctuations, and decadal Pacific basin variability [67]. ENSO causes low-frequency modulation of global atmospheric circulation, thereby impacting climate, including monsoons and hurricanes, as well as altering the productivity of oceans and ecosystems [68]. The effects of ENSO are evident on all the continents of the world. El Niño warms and dries the climate in Southeast Asia and Australia while it cools and moistens the southern states of America [69]. La Niña’s impacts are usually the opposite and can enhance monsoon rains in South Asia and Atlantic hurricane activity [69]. These climate variations affect agriculture, water resources, and the biosphere, making accurate ENSO predictions crucial for minimizing socioeconomic impacts [70]. Even though climate change may affect the ENSO dynamics, there is still inadequate information on whether the occurrence and intensity of ENSO have changed. The threat of more frequent extreme events underscores the need for further study and observation to improve our understanding of ENSO and its relationship to a warming climate [14,46,56].

#### ENSO phases and impact on Thailand

To understand the impact on Thailand, this study collected observational data on monthly precipitation (PPT), maximum and minimum temperatures (T_max_ and T_min_), and relative humidity (R_H_) from the Thai Meteorological Department from 1990 to 2024. The ENSO anomaly was collected from the National Oceanic and Atmospheric Administration’s Climate Prediction Center (Table 2). To classify an El Niño or La Niña phase, the threshold must be reached for at least six consecutive months from May to November, and the three-month running means must overlap the seasons [49,71].

**Table 2:** ENSO regions with spatial domain and data source [71].

**Region**	**Spatial domain**	**Data source**
NIÑO3.4	(5S-5N,170W-120W)	https://www.cpc.ncep.noaa.gov/data/indices/sstoi.indices
NIÑO3	(5N-5S, 150W-90W)	
NIÑO4	(5N-5S, 160E-150W)	
NIÑO1+2	(0-10S, 90W-80W)	

**Fig. 1:**
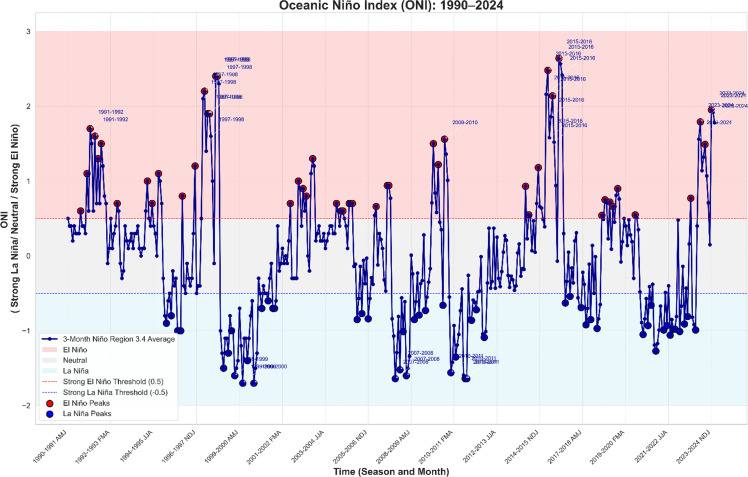
The ONI from 1990 to 2024 shows 3-month Niño Region 3.4 averages, highlighting El Niño (red), Neutral (grey), and La Niña (blue) phases with thresholds of ±0.5. Season labels mark intense events (|ONI| > 1.5), and red and blue circles indicate peaks. Neutral, Weak La Niña (WL), Strong La Niña (SL), Moderate El Niño (ME), Strong El Niño (SE), Very Strong El Niño (VSE), and Moderate La Niña (ML)).

Fig. 1 illustrates the monthly SST anomalies for 1990-2024, along with the specific events of ENSO (Neutral, Weak/Moderate/Strong El Niño, and La Niña). Some characteristics are detected during ENSO extremes, for example, the VSE in 1997-1998 (+2.4°C, SON) and 2015-2016 (+2.64°C, NDJ), with more decisive warming phases. On the other hand, marked cooling is observed during strong La Niña, for instance, 1998-1999 (-1.6°C, NDJ) and 2010-2011 (-1.64°C, NDJ). Thus, a moderate El Niño characterized moderate, milder SST anomalies in 2002-2003, and a Weak La Niña in 2022-2023. Neutral years exhibit small fluctuations, which do not exceed ±0.5°C, indicating stable climate conditions. The dataset suggests that ENSO is cyclic, and sharp fluctuations between warm and cold states have a significant impact on the world’s climate. The observations in 2023-2024 indicate the continuation of a strong El Niño at a temperature of +1.95°C, as of NDJ.

**Fig. 2:**
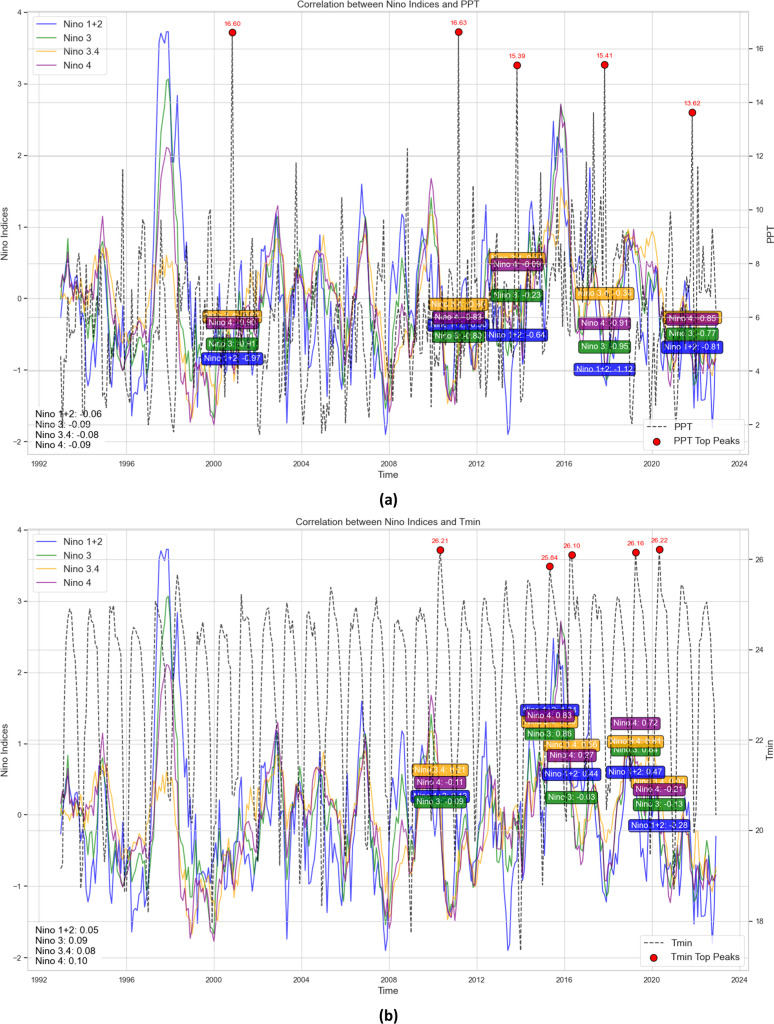

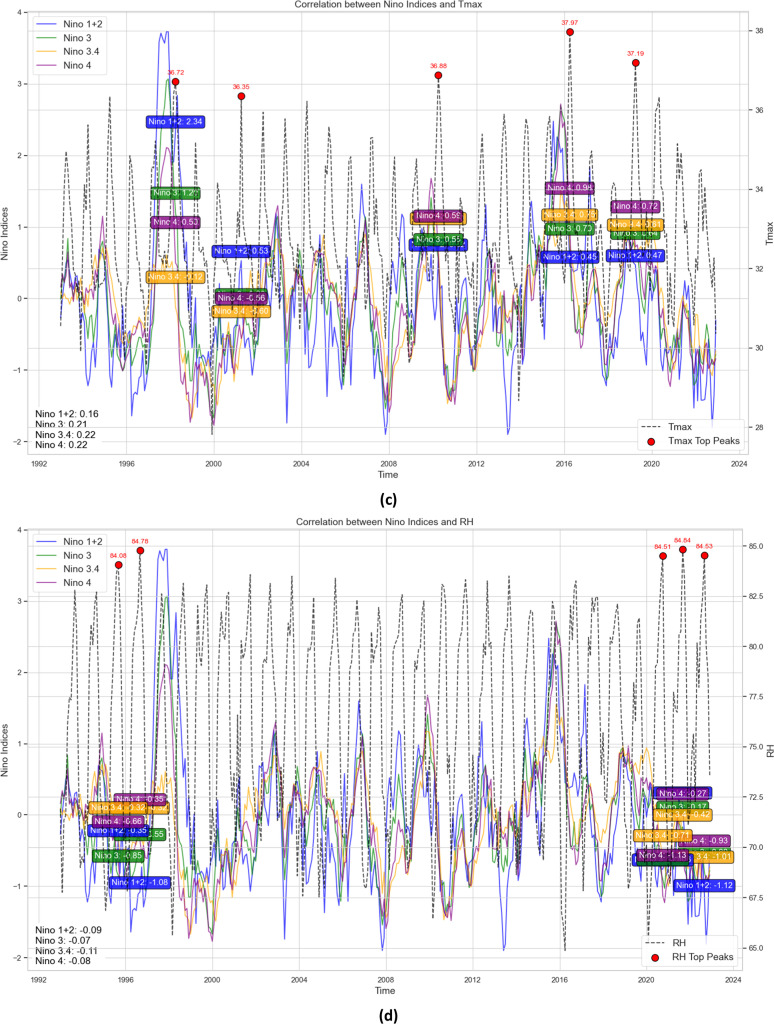
Regression analysis of Niño indices (Niño 1+2, Niño 3, Niño 3.4, Niño 4) and climate variables (a) PPT (mm), (b) Tmin (°C), (c) Tmax (°C), and (d) RH ( %) for the period 1990 to 2024. The plot also displays the Niño indices and the corresponding top peaks of each climate variable, along with their annotated values. The red dots indicate the significant peaks and their relationship to Niño indices.

Fig. 2 (a-d) The regression analysis of Niño indices (Niño 1+2, Niño 3, Niño 3.4, Niño 4) with climate variables (PPT, R_H_, T_max_, and T_min_) from 1990 to 2024 shows that the correlation is low, but not negligible. PPT exhibits weak inverse correlations with all Niño indices (e.g., Niño 3.4). The data also show that during positive Niño phases, rainfall decreased, which can be attributed to the weakening of monsoonal circulation. The correlation between the top PPT peaks and Niño indices highlights ENSO’s control over extremes (Fig. 2(a)). The R_H_ correlates weakly but negatively with Niño indices (e.g., Niño 3.4). The negative values of 0.016 and 0.011 in the El Niño phases are also indicated by the regression coefficients of the SSTA of the SHLT, suggesting decreased moisture availability due to subsidence and drying in ENSO-affected regions. Niño 3.4 has a comparatively higher correlation, providing convincing evidence for its role in variability associated with ENSO (Fig. 2(d)). Similar trends for T_max_ and T_min_ were observed (Fig. 2 (b and c)). As Niño indices are part of climate change, other atmospheric and oceanic factors may also play a role; therefore, a multivariate analysis is necessary to understand the regional climate better.

**Fig. 3:**
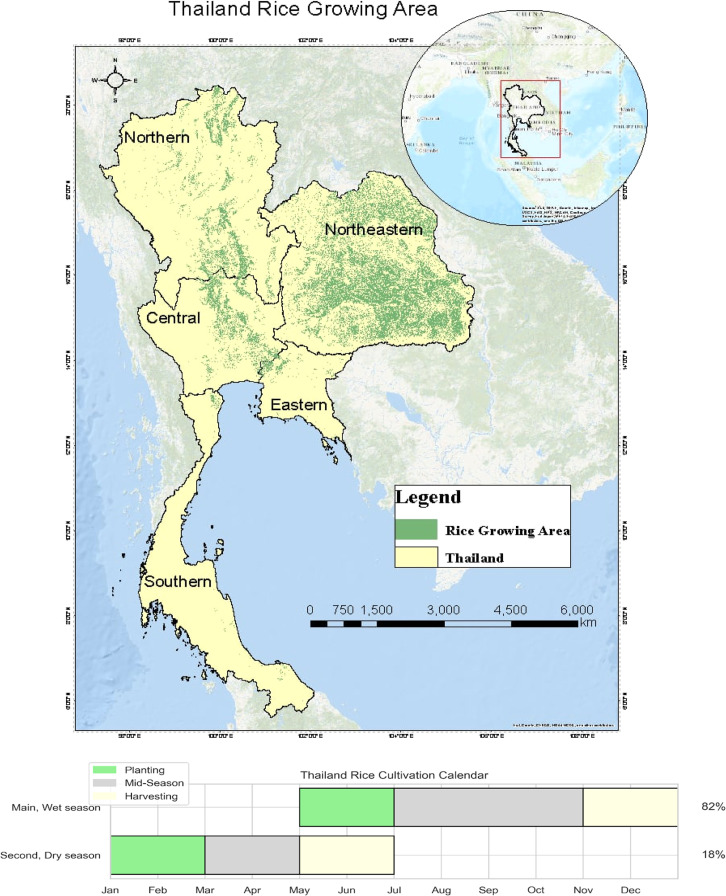
Rice cultivation area and rice growing calendar for Thailand.

Fig. 3 shows Thailand’s rice cultivation area and crop cultivation calendar [109]. Jasmine rice, the most cultivated strain, has a lower yield but remains a staple export, positioning Thailand among the world’s leading rice exporters [110]. Rice farming is water-intensive, requiring 1,500 cubic meters of water per rai, with the average farm size being 1.6 hectares per family [111]. U. S. Department of Agriculture statistics show rice production from 2014/2015 to 2024/2025 in terms of area harvested, milled production, rough production, and yield. The area harvested also showed variations, ranging from 9,444 thousand hectares in the 2015/2016 fiscal year to 11,072 thousand hectares in the 2022/2023 fiscal year. The average harvested area for the five years (2019/20–2023/24) was 10,565 thousand hectares, which is only 1 % above the LTA. Milled production improved performance from 15,800 thousand tons in 2015/2016 to 20,909 thousand tons in 2022/2023, slightly above the 5-year moving average of 19,461 thousand tons [112]. Rough production also followed the milled production pattern, reaching 31,680 thousand tons in 2022/2023, which is 56 % more than the 5-year average of 29,486 thousand tons. Yield also showed a progressive trend, increasing from 2.53 tons/ha in 2015/2016 to 2.90 tons/ha in 2017/2018, with recent yields slightly declining to 2.85 tons/ha. These improvements are attributed to advancements in farming techniques, the use of improved seeds, and the efficient utilization of resources. The dataset shows that productivity has been improving steadily and points to 2022/2023 as the year of high performance, a breakthrough point in agricultural efficiency [112].

### Case studies on ENSO impacts on rice yield in Thailand

Thailand’s climatic conditions include monsoons, intertropical convergence zones, and tropical cyclones, which exhibit high interannual variation [71,72]. Rainfed rice, which accounts for 65 % of Thailand’s total rice-growing area, is most affected by this variability [73]. ENSO events act as a climatic oscillation that increases climatic variability by producing severe droughts or floods during the critical growing seasons [21]. El Niño normally suppresses the wet season, leading to water shortages, while La Niña increases rainfall, which may cause floods [13,21].

Studies from the last few decades support the notion that ENSO influences rainfall and rice yield in Thailand [[72], [73], [74]]. Jintrawet and Buddhaboon (2012) utilized rainfall and rice yield data from 1980 to 2002 to demonstrate that El Niño reduced total annual rainfall but did not consistently decrease primary-season rice yields at the provincial level. This finding suggests that regional characteristics, such as irrigation and soil conditions, modify the relationship between ENSO and rice yield [73]. Wongkhunkaew et al. (2020) found similar trends in northeast Thailand, where SPEI affected rice production but not yields in a way [74]. Although water availability affects production areas, these differences suggest that yields depend on soil fertility and farm management practices [73,74].

ENSO-driven droughts affect water availability for agriculture and disrupt essential processes, including photosynthesis and transpiration [75,76]. For example, Setsungnern et al. (2023) found that the 2015 intense El Niño event resulted in an 18.3 % decrease in cumulative rainfall, a decrease in water levels in the paddies, and an increase in irrigation [75]. The study also indicated that GPP and net carbon budgets were lower during the El Niño year and were found to be in proportion to the 2.8–5.7 % decline in grain yield compared to the neutral years [75]. La Niña flooding also brings other issues, including enhanced methane emissions from saturated soil and changes in planting seasons. Such conditions make it challenging to manage farms because of the need for early drainage and pest control [113,114]. ENSO affects the country in varying degrees, as some areas are characterized by high terrain, diverse soil types, and varying irrigation levels [77,78]. Some regions that are most affected rely heavily on rain-fed agriculture, such as the northeast region. These areas often experience delayed planting and lower yields during El Niño years.

On the other hand, the irrigated areas in the central part of the country are more stable and can produce the same yields even in climatic fluctuations [79].

El Niño events result in less precipitation, a problem for rice cultivation due to water availability issues in rain-fed systems in Thailand. In another study, Chaweewan and Ekasit (2010) found that El Niño reduces rainfall during critical crop-growing periods, leading to lower yields. Other indices, such as the Multivariate ENSO Index (MEI), show that water availability was significantly reduced during phases of El Niño in the Central Plain [78]. Analyses using the Multivariate ENSO Index (MEI) also show significant reductions in water availability during El Niño phases, especially in the Central Plain [78].

In contrast, La Niña events often bring increased rainfall and flooding, which can be beneficial and detrimental. Flooding during early growth phases may lead to crop failure, while increased water availability later in the season may boost productivity [80,81]. According to Wonlee (2018), April, May, and October are the transitional months most affected by ENSO in the eastern region of Thailand; therefore, water management is crucial during these months [80]. Felkner et al. (2009) coupled crop simulation models with economic analysis and forecasted yield losses of up to 25 % under extreme El Niño conditions due to water and heat deficits [82]. Regarding quantity, yields vary between 15 % and 30 %, depending on the phase of the ENSO. El Niño decreases yields by 15-20 %, while La Niña occasionally increases yields by up to 10 % [80,81]. DSSAT models also stress that water availability, a function of ENSO rainfall distribution, is a key determinant of yield results, suggesting the need for water forecasting systems [82].

Table 2 summarizes that the ENSO is a significant source of climate change affecting rice production in Thailand’s rain-fed rice systems. Improving irrigation systems, embracing climate-smart technologies, and utilizing more accurate forecasting tools will help protect Thailand’s rice sector from ENSO and other future climatic risks (Tables 3, 4 and 5).

**Table 3:** Different aspects of ENSO impacts (El Niño and La Niña) in Thailand.

**ENSO impacts**	**El Niño**	**La Niña**
Rainfall	Reduced rainfall by 18.3 % during intense events, leading to water deficits [75].	Increased rainfall often causes flooding and waterlogging [76].
Rice Yields	Rainfed crop yield decreased by 2.8–5.7 % due to water stress, reduced gross primary production, and carbon budgets [75].	Potential yield reductions from flooding and delayed planting schedules [74].
Carbon and Energy Fluxes	Lower methane emissions and reduced gross primary production; increased evapotranspiration during dry conditions [75].	Higher methane emissions from waterlogged soils; potential for anaerobic conditions in paddy fields [72].
Geographical Variability	More significant impact in rainfed regions such as the northeast due to limited water resources [73].	Flood-prone regions experience higher risks; irrigated areas are less affected [79,115]
Adaptation Strategies	Improved irrigation, adoption of drought-tolerant varieties, and crop calendar adjustments to align with rainfall patterns [72].	Enhanced drainage infrastructure and real-time monitoring to prevent waterlogging [73].
Policy Recommendations	Early warning systems, farmer education on drought management, and investment in resilient infrastructure [2].	Policies supporting flood management and recovery measures [2,115].

**Table 4:** ML and DL Models for Assessing ENSO Impacts: Technical Descriptions, Advantages, and Limitations.

**AI**	**Model**	**Technical Description**	**Advantages**	**Limitations**	**Sources**
Machine Learning	RF	An ensemble method using multiple decision trees to improve accuracy by averaging predictions or majority voting for classification.	Manages high-dimensional data, reduces overfitting, identifies feature importance, and supports classification and regression.	It struggles with imbalanced data and high memory usage and requires careful hyperparameter tuning.	[2,44,91]
	SVM	Finds the optimal hyperplane to separate classes, using kernel functions for linear and nonlinear problems effectively.	Effective for high-dimensional data, robust classification, and generalize well with the proper kernel.	Sensitive to kernel choice, computationally expensive, and prone to overfitting with small datasets.	[92],^93^].
	DT	Tree-structured models split data based on features; leaf nodes represent final predictions for classification or regression tasks.	Easy to interpret, supports categorical/continuous data, works on small datasets, and requires no scaling.	Greedy splitting can result in suboptimal trees, sensitivity to small data changes, and a lack of smooth predictions.	[91,94,95].
	GBM	Sequentially builds models to correct residual errors, combining weak learners for highly accurate predictions.	High accuracy, robust classification/regression, reduces overfitting and manages outliers well.	Requires careful parameter adjustment, limited interpretability, and struggles with overfitting unless finely tuned.	[96,97]
	KNN	Data points are classified based on the majority class among the closest data points, determined by a distance metric.	Easy to implement, no training phase, supports all data types and works well with small datasets.	Slow for large datasets, lacks robustness to noise, and requires an optimal value of ‘k.’	[88,98].
Deep Learning	CNN	Extracts spatial hierarchies in image-like data using convolutional layers and learnable filters for pattern recognition across scales.	Captures spatial features in images, reduces manual feature engineering, and performs well on grid-structured data.	Overfitting without regularization is computationally intensive and lacks interpretability for learned features.	[99,100]
	LSTM	Specialized RNN capturing long-term dependencies in sequential data through memory cells and gating mechanisms. Prevents vanishing gradient issues.	Oversees time-series data, preserves dependencies, is robust for sequential tasks, and is effective for forecasting.	Gradient explosion risks are computationally expensive and require substantial training data for generalization.	[44,71,101,102]
	BNNs	Incorporates probabilistic methods into neural networks, representing weights as distributions to quantify prediction uncertainty in tasks requiring reliability.	Estimates uncertainty, prevents overfitting, and is suitable for decision-making in uncertain environments.	Limited scalability, complex posterior computations, and computational overhead with high-dimensional data.	[103,104]
	Autoencoders	Compresses and reconstructs data for unsupervised feature extraction or dimensionality reduction, with a focus on key representations within bottleneck layers.	Effective for anomaly detection, feature extraction, and dimensionality reduction of high-dimensional data.	Struggles with high noise levels lack interpretability and require careful architectural design to avoid trivial reconstructions.	[[106], [107], [108]].
	DNN	Multilayer architecture can learn hierarchical, nonlinear relationships in data for complex tasks like classification, regression, and forecasting.	It captures nonlinear relationships and is flexible and powerful for large-scale problems.	Prone to vanishing gradients requires large training data and extensive tuning for optimal performance.	[107,108].

**Table 5:** ML and DL methods applied to assess the impact of ENSO on different crop yields.

**ML/DL** **Method**	**Data type and duration**	**Crop**	**Evaluation criteria**	**Study results**	**Limitations**	**Reference**
RF	Climate indices and spring rainfall data (1985–2016)	Wheat	R², accuracy metrics	RF models explained 33–66 % of the variation in wheat yield using ENSO-related indices and forecasted rainfall. The most influential predictors were SOI and forecasted spring rainfall in eastern Australia.	Limited to regional data; lacks generalizability to other crops or regions.	[94]
GBM	CMIP6 climate data (1980–2014)	Wheat and Rice	Bi-wavelet coherence, Mann-Kendall test	TN10p and WSDI showed strong associations with wheat and rice yields, respectively. GBM projected future temperature extremes under SSPs, indicating spatial variations in DTR and other indices.	Rely on climate projections and uncertainties in future climate scenarios.	[123]
D-Vine Copula Regression	Climate mode indices (1983–2013)	Wheat	Quantile regression, cross-validation	D-Vine models accurately captured the dependence between climate indices and wheat yield. A significant negative correlation with IOD was observed during the early growth phases.	Limited to linear dependencies may miss complex nonlinear interactions.	[121]
ANNs	ENSO-related indices (1900–2018)	Wheat	RMSE, correlation	ANN models outperformed traditional models in forecasting spring rainfall and were practical for predicting wheat yields in Queensland and New South Wales.	Requires large datasets; prone to overfitting without proper validation.	[124]
CSM-CROPGRO-Cotton Model	Weather data (temperature, precipitation) for 97 counties, 38–107 years (1900–2006)	Cotton	RMSE, Willmott Index, Modeling Efficiency (ME), R²	ENSO phase impacts on cotton yield varied by planting dates. La Niña favored higher yields for early planting (before May 9), while El Niño favored later planting (after May 23).	Limited to historical data; may not account for future climate variability.	[59]
Linear Correlation/Regression Analyses	National and state wheat yield data (1948–2013), SSTs (1950–2011)	Wheat	Linear correlation, regression, bootstrap method, p-value	ENSO and IOD indices have a major influence on wheat yield anomalies. El Niño reduces yield, while La Niña increases it. IOD and ENSO Modoki also exhibit distinct teleconnections that affect yields.	Simplistic approach may overlook nonlinear relationships between ENSO and yields.	[122]
Synthetic Analysis and Bootstrap	Crop census data (∼12,000 political units, ∼30 years), ENSO indices	Maize, Rice, Wheat, Soybean	Statistical significance (p-value), R², yield anomalies	ENSO phases significantly influenced global crop yields: El Niño reduced yields of wheat, rice, and maize, but increased soybean yields; La Niña had the opposite effect on wheat and rice.	Aggregated data may mask local variability, which is limited by the data resolution.	[62]
SVM and LSTM	Weekly and monthly meteorological data (1982–2021)	Rice	Correlation (r), and RMSE	LSTM outperformed SVM; the highest accuracy was achieved with weekly datasets using eight variables (temperature, precipitation, etc.) from downscaled data.	Computationally intensive; requires high-quality, high-resolution input data.	[92]
Ridge Regression, RF, Gradient Boosted Trees, ANN, LSTM	Municipality-level yield data, climate data, and remote sensing data (2001–2020)	Soybean	Relative RMSE and R²	The ensemble model and ANN achieved the best performance, with an RRMSE of 6 % at the national level, thereby enhancing accuracy closer to harvest time.	Limited by data availability; may not generalize to other crops or regions.	[125]
BNN	ENSO Oceanic Index data (1–25 months before harvest), cacao yield data (2013–2018)	Cacao	MSE, R², and Cross-validation error	BNN explained up to 77 % of yield variation using long-term ENSO profiles; short-term models were simpler but slightly less accurate (69 %). Fertilizer response varied across ENSO profiles.	Limited to cacao; lacks validation for other crops or regions.	[126]

The literature review highlights the impact of the ENSO phenomenon on the Thai climate and agriculture, particularly rice, which is rain-fed. The climatic variability resulting from ENSO, such as El Niño droughts and La Niña-induced floods, exacerbates challenges to sustainable agriculture in the region. Climatic records from 1990 to 2024 indicate that phases of El Niño are associated with changes in precipitation, while phases of La Niña are characterized by increased precipitation. These changes are crucial in determining water distribution during the critical growth phases, which significantly impact rice production. Nevertheless, the degree of these effects is conditioned by regional characteristics, such as irrigation facilities, soil conditions, and farm practices, which result in spatial variability in yield outcomes. Additionally, ENSO events impact critical physiological processes, including photosynthesis and transpiration, thereby limiting crop production. The results underscore the need for improved predictive models and more adaptable approaches, including efficient water management and climate-resilient farming techniques, to mitigate the impact of ENSO on Thailand’s agriculture as climate volatility intensifies.

### AI-based techniques for assessing ENSO impacts on crop yield

This section evaluates AI-based methodologies for quantifying the impact of ENSO phases on crop yields. Given the scarcity of studies specific to Thailand that explore the application of AI in assessing the effects of ENSO on agricultural productivity, a broader perspective is adopted. To provide comprehensive insights and identify transferable methodologies, this section incorporates findings from global studies, highlighting their relevance and potential applicability to the Thai context.

Prediction models are categorized broadly into dynamical, statistical, and hybrid models [83]. Dynamical models, physically based models, employ partial differential equations to model atmospheric, land, and ocean processes [84]. These models have been enhanced with the aid of increased computational capabilities and the availability of remote sensing data [85], yet they still have limitations due to their sensitivity to initial conditions and the inherent stochasticity of climate systems [44,83]. The statistical models rely on past data to establish the relationship and depend on indicators [86]. These are autoregressive models, regression, and Bayesian analysis [87]. However, statistical models are usually efficient and straightforward but are based on linear structures and cannot be applied to complex and nonlinear systems [88]. Hybrid models combine dynamic and statistical models, producing separate predictions using each technique and combining the results for enhanced accuracy [89]. This approach can potentially mitigate the issue of regional variability in predictors and improve lead times; however, it still requires further enhancement [83]. In recent years, new directions in AI and ML, like deep generative models and transfer learning, have offered the potential to improve prediction and uncertainty.

**Fig. 4:**
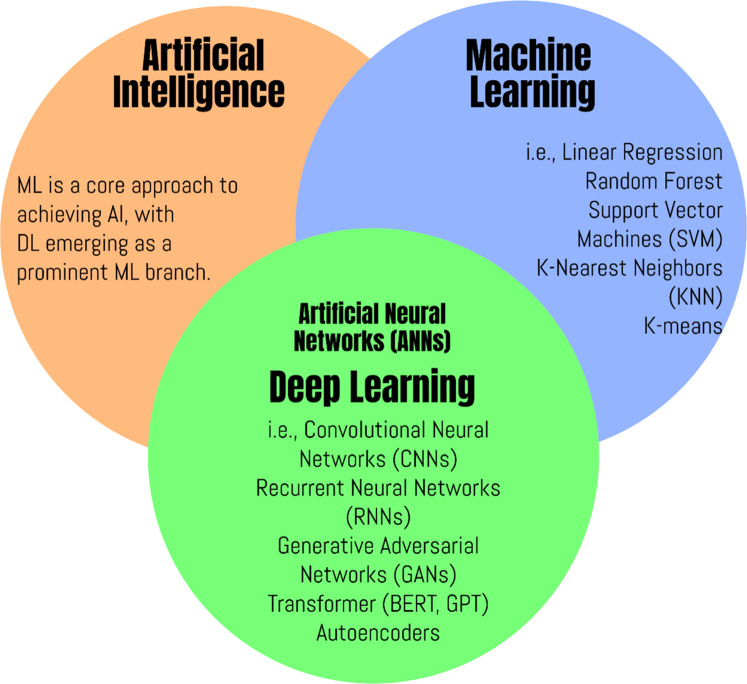
The connection between AI, ML, and DL.

The concept of AI, introduced at the Dartmouth Conference in 1956, aims to enable machines to think and respond like humans [90]. ML is a core approach to achieving AI, with DL emerging as a prominent ML branch [44] (Fig. 4). Proposed by Hinton et al. in 2006, DL leverages multi-layered neural networks for automatic feature extraction, with deeper networks offering superior feature representation for complex tasks [90]. Artificial neural networks (ANNs) and support vector machines (SVMs) have enhanced the capability to model nonlinear relationships, but these models are prone to overfitting and require substantial data for training [44]. ML and DL have recently been effectively utilized for meteorological and climate predictions, including ENSO events [56].

The application of AI, ML, and DL in assessing the effects of ENSO on cereal crop yield has been found to improve the accuracy of predictions under climate change. The most used models are Random Forest (RF), Support Vector Machines (SVM), Decision Trees (DT), Gradient Boosting Machines (GBM), and K-Nearest Neighbors (KNN) due to their ability to classify or predict crop yields using environmental factors affected by ENSO events.

RFs are ML models that combine outputs of multiple DTs constructed using random data set samples [44]. This model is advantageous when dealing with large datasets, and it can give feature importance, which is very useful in determining which climatic factors are most important in crop production [2]. However, RF models face computational challenges with large datasets and are also criticized for being ‘black box’ models, as the output of many trees is difficult to interpret [91]. Like DT, SVM is suitable for high-dimensional data and identifying the appropriate hyperplanes for classification, which is essential when evaluating the effects of ENSO on crop production [92]. However, SVM is sensitive to the choice of kernel and its parameters, and it is not well-suited for noisy or outlier data.

Additionally, its scalability for large datasets is a problem [93]. DTs are easy-to-use and powerful models that split data into branches based on feature tests and make the final decision at the end nodes [91]. The main strength of the DT is that it is relatively simple to apply and explain the interaction between climatic factors related to ENSO and crop yields [94]. However, DT models are known to overfit, which can occur when the dataset is small or noisy, thereby impacting the generality of the results [95]. GBM is a boosting algorithm where new models are built sequentially, and the errors from the previous model are minimized, making it highly accurate and relatively insensitive to outliers [96]. However, using GBM models may be slow and sensitive to the choice of hyperparameters, especially when data are noisy [97]. Finally, the KNN is a non-parametric technique that uses nearest neighbors for classification [88]. KNN is easy to implement and computationally efficient when the dataset size is small, and it can handle various types of data; however, it is susceptible to noise and exhibits high time complexity when the dataset size is large [98].

DL techniques, such as Convolutional Neural Networks (CNNs), Recurrent Neural Networks (RNNs), Long Short-Term Memory (LSTM) networks, Bayesian Neural Networks (BNNs), and Deep Neural Networks (DNNs), have been applied to analyze climate and crop data to identify nonlinear relationships and temporal dependencies.

CNNs work best when applied to spatial data, such as satellite images of crop fields, since they learn a spatial hierarchy through convolutional layers [99]. This ability makes CNNs ideal for identifying spatial patterns of ENSO-related impacts on crop yields [99]. However, CNNs require a large set of labelled data for training and can be computationally expensive, making them unsuitable for large geographical areas or time series data [100].

RNNs, particularly LSTMs, are popular for time series forecasting because they can model the temporal dependencies in the climate data caused by ENSO events [71,101]. LSTMs are most valuable when forecasting yields influenced by long-term climatic changes because they eliminate the vanishing gradient issue and maintain helpful information over time [102]. However, LSTMs are computationally expensive and have numerous hyperparameters to tune, making them unsuitable for use in low-resource environments [44]. The BNNs incorporate probabilistic methods, allowing the model to express uncertainties in its predictions, which is beneficial for decision-making under climate variability [103]. Although BNNs can express uncertainty, they are computationally intensive, and prior modeling is a crucial step that makes their implementation difficult in complex problems [104]. Autoencoders, used for unsupervised learning, are good at feature extraction and identifying outliers [90].

Regarding ENSO and crop production, autoencoders can simplify climate and crop yield data, thereby identifying outliers that may indicate the impact of ENSO [105]. Nonetheless, autoencoders are known to overfit the training data when no form of regularization is applied, and the model’s performance strongly relies on the size of the dataset [106]. DNNs are extended networks containing multiple layers of neurons and can capture nonlinear dependencies in the data, making them suitable for capturing crop production under ENSO impacts [107]. While DNNs can model complex data patterns and have broad applications, the training process is computationally intensive [108].

#### Case studies and applications of AI

The use of AI methods, including ML and DL, for evaluating the impacts of ENSO on cereal crop production represents a transformative approach in agricultural research. Traditional statistical models, while helpful, often fall short of capturing the complex, nonlinear relationships between climatic variables and crop yield dynamics. The integration of AI addresses these limitations by leveraging advanced algorithms that can process large datasets with high temporal and spatial resolution.

Zennaro et al. (2021) investigated ML for climate-related risk assessments, identifying decision trees, random forests, and artificial neural networks (ANNs) as the most frequently applied approaches [116]. Gibson et al. (2021) compared ML models to dynamical models for predicting large-scale rainfall patterns in the United States (1980–2020), finding that ML methods can match or surpass traditional downscaling models [117]. Similarly, Rampal et al. (2022) demonstrated the superior performance of Convolutional Neural Networks (CNNs) in precipitation downscaling over New Zealand, especially for extreme events [118]. Ham et al. (2019) also highlighted the effectiveness of CNNs in forecasting ENSO events, outperforming existing dynamical models and offering valuable insights into ENSO’s complex mechanisms [119]. Using an agroclimatic model, Potgieter et al. (2005) classified El Niño years into three distinct patterns affecting wheat yields in the Australian wheat belt. The detrended yield-SOI relationship highlights the influence of ENSO on agriculture [120]. Nguyen-Huy et al. (2018) revealed that Indian Ocean variability significantly impacted wheat yields in most Australian states, with Pacific Oceanic conditions having a more substantial influence in Queensland [121]. These findings align with Yuan and Yamagata (2015), who attributed ENSO impacts to baroclinic effects in Queensland and Rossby wave-induced moisture changes elsewhere [122].

Furthermore, Table 3 highlights the application of ML and DL techniques in predicting ENSO impacts on crop yields. These methods utilize diverse datasets and metrics to address nonlinear relationships between climatic indices and crop yields. RF and GBM effectively predict wheat and rice yields, with RF models explaining 33–66 % of the variability in wheat yields in Australia. Advanced techniques, such as D-Vine Copula Regression, model dependencies like the IOD’s influence on wheat growth. ANN, SVM, and LSTM outperform traditional models, with LSTM demonstrating the best performance in rice yield prediction. Emerging approaches, such as BNN, enhance modeling accuracy, confirming the potential of ML/DL in climate-crop yield assessments.

### Discussion

**RQ-1:** Influence of ENSO Phases on Thailand’s Climate Variability and Crop Productivity

ENSO phases have a profound influence on Thailand’s climate, affecting precipitation and temperature patterns. El Niño events are associated with reduced rainfall, leading to drought conditions, while La Niña events typically result in increased precipitation, often causing floods [3,4]. These climatic extremes disrupt water availability, which is critical for crop growth, particularly in rainfed agricultural systems that dominate Thailand’s rice production [2,73].

During El Niño, the weakening of the Walker Circulation and easterly trade winds leads to above-average SSTs in the eastern Pacific, suppressing monsoon activity in Southeast Asia [64]. It results in significant water deficits during the critical growth stages of rice, reducing yields by 15-20 % [80,81]. Conversely, La Niña enhances the Walker Circulation, increasing rainfall and often causing waterlogging and flooding, which can delay planting and reduce yields by up to 10 % [74]. Neutral phases, characterized by minimal SST anomalies, typically result in more stable climatic conditions, enabling more predictable agricultural outcomes [2,76].

The regional variability in Thailand further complicates the impact of ENSO. For example, the northeastern region, which relies heavily on rainfed agriculture, is more susceptible to El Niño-induced droughts, while the central region, with its extensive irrigation infrastructure, is more resilient [79]. This spatial heterogeneity highlights the need for localized adaptation strategies to mitigate the impacts of ENSO.

**RQ-2:** Specific Effects of ENSO-Induced Climate Variability on Rice Yields in Thailand

Rice, a water-intensive crop, is particularly vulnerable to ENSO-induced climate variability. The review highlights that El Niño events reduce cumulative rainfall by 18.3 %, leading to significant water stress and reduced GPP in rainfed rice systems [75]. This water deficit affects critical physiological processes such as photosynthesis and transpiration, reducing grain yields by 2.8-5.7 % compared to neutral years [75]. La Niña, on the other hand, often results in excessive rainfall, leading to flooding and waterlogging. These conditions can delay planting schedules and increase methane emissions from waterlogged soils, further complicating farms [72,113,127]. Despite the potential for increased water availability, the timing and intensity of La Niña-induced rainfall often do not align with the critical growth stages of rice, leading to suboptimal yields [80].

The impact of ENSO on rice yields is also influenced by regional characteristics such as soil type, irrigation infrastructure, and farm management practices [79]. This variability underscores the importance of adaptive strategies, such as improved irrigation systems and drought-tolerant crop varieties, in enhancing resilience to ENSO-induced climate variability [72,109].

**RQ-3:** Application of AI Methods in Assessing ENSO Impacts on Cereal Crop Production

Integrating ML and DL techniques offers a transformative approach to understanding and predicting the impacts of ENSO on cereal crop production. Traditional statistical models, while helpful, often fail to capture the complex, nonlinear relationships between climatic variables and crop yields [54]. AI methods, however, excel in processing large datasets with high temporal and spatial resolution, making them well-suited for capturing the intricate dynamics of ENSO impacts [56]. The review studies in this section underscore that AI-based techniques, including ML and DL, provide significant advancements in quantifying the impacts of ENSO on crop yields, thereby overcoming the limitations of traditional models. While dynamical and statistical models remain valuable, AI methods can better manage nonlinear relationships and complex climate data. ML models like RF, SVM, and GBM are commonly used for classification and regression tasks, offering robust performance despite noise and significant dataset challenges. DL models, such as CNN and LSTM networks, enhance predictions by modeling spatial and temporal dependencies inherent in ENSO-related impacts. Despite their computational intensity, these models can provide more accurate, scalable, and interpretable predictions when managing large, high-dimensional datasets. Further research and optimization of these models will be crucial for improving the accuracy and applicability of ENSO-related yield forecasting in Thailand’s agricultural sector.

## Conclusion

ENSO originates in the tropical Pacific Ocean, and its global impacts lead to severe droughts, floods, and a reduction in crop productivity across many regions, including Thailand. Therefore, understanding and predicting the impact of ENSO on crop productivity is critical.

This study critically analyzes key studies published over the past two decades. This review has examined the interplay between ENSO and cereal crop yields, with a primary focus on Thailand’s rice production as a case study. The analysis reveals that ENSO phases have a significant impact on rice yield, with El Niño events leading to reduced rainfall and drought conditions, while La Niña phases result in high rainfall and potential flooding. These climate extremes disrupt critical growth periods for rainfed rice, impacting yields and food security in Thailand.

Furthermore, due to the limited number of case studies on AI-based methods for ENSO impact assessment on crop productivity in Thailand, this review considered case studies from a global perspective. These techniques have shown promise in improving the precision of ENSO-related agricultural forecasts. In selected case studies, models have efficiently managed complex, nonlinear data relationships and shown promising results in capturing the impact of ENSO on various crops.

Future studies should focus on creating combined AI and physical models to enhance prediction accuracy and scalability. Better data sets that incorporate regional differences and extend climate change predictions are critical to improving AI outcomes.

## Limitations

This review has some limitations. First, the current review is divided and region-specific, lacking a global assessment of ENSO’s asymmetrical impact on crop yield. Second, the variability in regional conditions, such as irrigation type and infrastructure, soil types, and farm practices, presents complexities that current AI-based models cannot fully address.

## List of abbreviations

**Table:** 

AI	Artificial Intelligence
ANN	Artificial Neural Network
ARIMA	AutoRegressive Integrated Moving Average
BNN	Bayesian Neural Network
CNN	Convolutional Neural Network
CV	Coefficient of Variation
DL	Deep Learning
DSSAT	Decision Support System for Agrotechnology Transfer
DT	Decision Tree
ENSO	El Niño-Southern Oscillation
GBM	Gradient Boosting Machine
GPP	Gross Primary Production
IOD	Indian Ocean Dipole
LSTM	Long Short-Term Memory
MEI	Multivariate ENSO Index
ML	Machine Learning
NOAA	National Oceanic and Atmospheric Administration
NMME	North American Multi-Model Ensemble
ONI	Oceanic Niño Index
PPT	Precipitation
RF	Random Forest
RH	Relative Humidity
RMSE	Root Mean Square Error
RNN	Recurrent Neural Network
SD	Standard Deviation
SSTA	Sea Surface Temperature Anomaly
SVM	Support Vector Machine
Tmax	Maximum Temperature
Tmin	Minimum Temperature
USDA	United States Department of Agriculture
VSE	Very Strong El Niño

## Ethics statements

No data was used in this research.

## Funding

No Funding was used in this research.

## Declaration of Competing Interest

The authors declare that they have no known competing financial interests or personal relationships that could have appeared to influence the work reported in this paper.
